# From Chondrocytes to Chondrons, Maintenance of Phenotype and Matrix Production in a Composite 3D Hydrogel Scaffold

**DOI:** 10.3390/gels8020090

**Published:** 2022-02-02

**Authors:** Mahmoud Amr, Alia Mallah, Samina Yasmeen, Bernard Van Wie, Arda Gozen, Juana Mendenhall, Nehal I. Abu-Lail

**Affiliations:** 1 One UTSA Circle, Department of Biomedical Engineering and Chemical Engineering, The University of Texas at San Antonio, San Antonio, TX 78249, USA; mahmoud.amr@my.utsa.edu (M.A.); alia.mallah@utsa.edu (A.M.); 2 Department of Chemistry, Morehouse College, Atlanta, GA 30314, USA; samina.yasmeen@morehouse.edu (S.Y.); juana.mendenhall@morehouse.edu (J.M.); 3 Gene and Linda Voiland School of Chemical Engineering and Bioengineering, Washington State University, Pullman, WA 99164, USA; bvanwie@wsu.edu; 4 School of Mechanical and Materials Engineering, Washington State University, Pullman, WA 99164, USA; arda.gozen@wsu.edu

**Keywords:** articular cartilage, biomaterials, chondrons, hydrogels, osteoarthritis, tissue engineering

## Abstract

Osteoarthritis (OA) is a degenerative disease characterized by articular cartilage (AC) degradation that affects more than 30 million people in the USA. OA is managed with symptom-alleviating medications. Matrix-assisted autologous chondrocyte transplantation (MACT) is a tissue-engineered option, but current products are expensive and lack mechanical tunability or processability to match defect mechanical properties and anatomical shapes. Here, we explore the efficacy of a biocompatible hydrogel-based scaffold composed of sodium alginate, gelatin, and gum Arabic—referred to by SA–GEL–GA—to support bovine articular chondrocyte (bAChs) proliferation, pericellular matrix (PCM), and extracellular matrix (ECM) production. bAChs were grown for 45 days in SA–GEL–GA. Their viability, their live/dead status, histological staining, biochemical assays for glycosaminoglycans (GAGs) and collagen, atomic force microscopy (AFM) imaging, and immunofluorescence staining of collagen I, collagen II, aggrecan, and CD44 were assessed. We found that SA–GEL–GA was not cytotoxic, induced cellular proliferation by 6.1-fold while maintaining a round morphology, and supported ECM deposition by producing 3.9-fold more GAG compared to day 0. bAChs transformed into chondrons and produced a PCM enriched with collagen II (3.4-fold), aggrecan (1.7-fold), and CD44 (1.3-fold) compared to day 0. In summary, SA–GEL–GA supported the proliferation, ECM production, and PCM production of bAChs in vitro.

## 1. Introduction

Articular cartilage (AC) is a tissue that lines articulating joints such as fingers, hips, and knees. AC provides lubrication between joints. In the knee, AC plays a critical load-bearing role, where it needs to support the body’s weight as well as withstand excessive forces experienced in daily tasks such as walking or running [1]. AC is a low cellular density tissue with only 2% specialized cells called chondrocytes, that are responsible for the maintenance of its extracellular matrix (ECM) throughout a person’s life. Chondrocytes are embedded in an ECM that is composed of 80% water and 20% solid components. The solid part of the ECM is made of collagens (mainly collagen II) and proteoglycans (mainly aggrecan and chondroitin sulfate) [2]. Within the ECM, chondrocytes exist as a metabolic building unit called a “chondron”. A chondron is made of one to 8 chondrocytes, surrounded by a specialized matrix called pericellular matrix (PCM) enriched with collagen II, aggrecan, and collagen VI. The PCM is then surrounded by a territorial matrix (TM), and a further interterritorial matrix (IM) [3]. While the exact functions of these matrices are not very well understood, research has shown that they play a critical role in protecting chondrocytes from load-induced damage, as well as regulate molecular transport between cells [4].

AC functionality in the knee stems from its unique composition and structure, as it is composed of four different zones with different mechanical properties, fibril alignments, cellular morphologies, and biochemical compositions [2]. These zones are: (1) the superficial zone, (2) interstitial or intermediate zone, (3) radial or deep zone, and finally (4) the calcified zone. The superficial zone is made of flattened chondrocytes embedded in densely packed collagen II fibrils arranged parallel to the articulating surface that provides lubrication. The interstitial zone is mainly composed of negatively charged proteoglycans which play an important role in retaining water within AC, thus providing its load-bearing capacity due to the incompressibility of water [5]. To maintain the proper function of AC, homeostasis between catabolic and anabolic processes needs to be maintained, where chondrocytes constantly secrete matrix-building proteins and metalloproteinases that help in matrix remodeling and turnover. The disturbance of such homeostasis indicates the presence of a disease state.

Osteoarthritis (OA) is a degenerative disease that affects more than 32.5 million adults in the USA alone [6]. OA poses a huge socioeconomic burden with an approximate cost of $186 billion per year [7]. OA is a multifaceted disease that affects the whole joint in which AC tissue degradation and synovial inflammation are observed [8]. There currently exists no disease-modifying treatment for OA. The disease is managed using anti-inflammatory and hyaluronic acid injections, pain killers, and eventually patients need a total knee replacement (TKR) surgery [9].

Depending on the stage at which OA is detected, other treatment options with short-term success are possible, such as abrasion arthroplasty, where surgeons resurface the bone and stimulate stem cell migration to the defect site to initiate tissue growth. However, the tissue they create is a fibrocartilage that is mechanically inferior to AC and ends up failing from loads experienced by the joint [10]. An improvement to abrasion arthroplasty was introduced in 1994 in Europe. This technique is called matrix-assisted autologous chondrocyte transplantation (MACT). In MACT, chondrocytes are isolated from a patient’s own AC tissue, expanded in vitro on tissue culture plastic (TCP), then transplanted with the help of a three-dimensional (3D) scaffold into defect [11]. A few products have made it through the Food and Drug Administration (FDA) approval process, such as NeoCART^®^, NovoCART^®^3D, and CaReS^®^ [12,13,14]. All of these utilize collagen-based scaffolds, which are expensive. They also lack the mechanical tunability of scaffolds to match different zonal mechanical properties of native healthy AC and the processability to match the anatomical shape of a given defect. In this work, we evaluated the efficacy of a tricomponent biocompatible hydrogel-based scaffold we developed previously [15] for bovine articular chondrocyte (bAChs) proliferation, phenotype maintenance, and ECM and PCM deposition support in vitro. The scaffold is made of sodium alginate (SA), gelatin (GEL), and gum Arabic (GA), referred to as SA–GEL–GA, and can be crosslinked physically, ionically, and covalently. This scaffold offers an exceptional improvement in properties compared to the performance of its individual components used on their own, where gelatin suffers from poor mechanical properties and sodium alginate and gum Arabic suffer from poor cellular adhesion [16].

## 2. Results and Discussion

### 2.1. Cytotoxicity Evaluation

**Figure 1:**
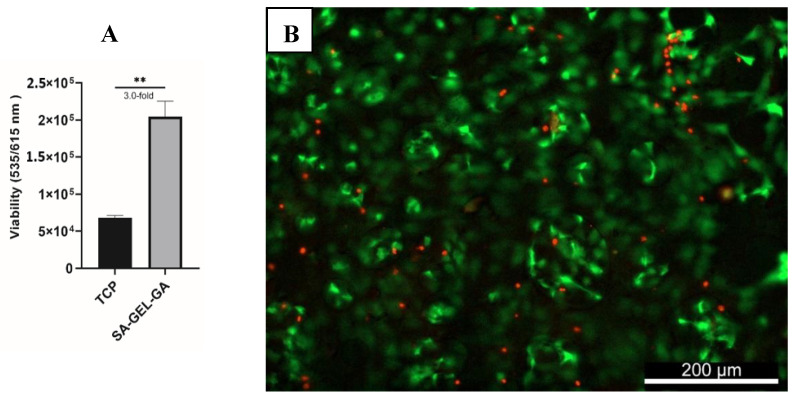
(**A**) Quantified viability of bAChs on SA–GEL–GA compared to TCP at day 45 as evaluated by PrestoBlue fluorescence assay at excitation/emission wavelengths (535/615 nm) (n = 3, mean ± standard error of the mean (SEM), ** *p* < 0.01). (**B**) Representative live/dead image of bAChs grown in SA–GEL–GA on day 45 using calcein-AM (green: live), and propidium iodide (dead: red), magnification: 10X.

It is crucial for any scaffold that will be used to support living cells in TE applications to be non-cytotoxic. SA–GEL–GA posed no cytotoxicity when investigated at day 45. bAChs cultured within SA–GEL–GA were three-fold more viable than bAChs grown on TCP (Figure 1A). This viability was confirmed qualitatively using calcein-AM and propidium iodide fluorescence labelling, as shown in the representative image of Figure 1B, where live cells were stained green and dead cells were stained red.

### 2.2. Cellular Distribution in Scaffolds and Cellular and Chondron Morphologies

**Figure 2:**
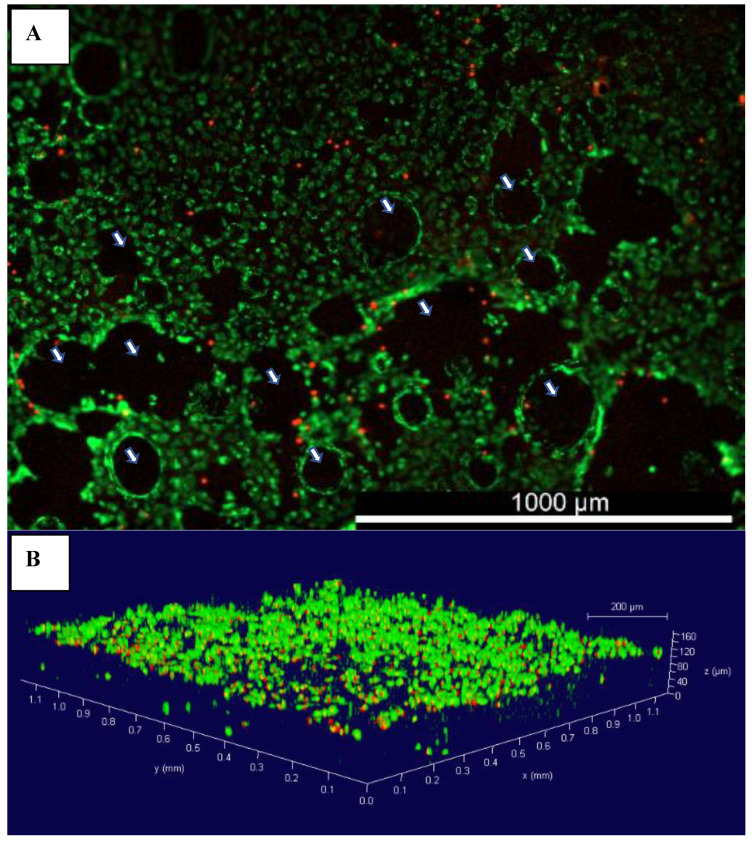
(**A**) Representative image of the distribution of bovine articular chondrocytes grown in SA–GEL–GA at day 45 imaged 1 h after incubation with membrane-specific DiOC6(3) dye and propidium iodide, magnification 4×; (**B**) 3D rendering of bovine chondrocytes grown in SA–GEL–GA using confocal microscopy; green is the cellular membrane, and red is the nucleus, magnification 10×;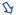 macropores.

The architectural aspect of an AC TE scaffold is of great importance, as it should provide enough porosity for cells to penetrate and migrate within the scaffold, as well as facilitate nutrients’ transport and waste exudation [17]. bAChs grown in SA–GEL–GA were homogenously distributed throughout the scaffold as indicated by membrane staining using DiOC6(3) (Figure 2A). The presence of macropores within tissues was also evidenced by the dark empty spots (Figure 2A) as indicated by the white arrows. The pores were randomly distributed within the gel and their distribution was not controlled. The 3D distribution was visualized using confocal 3D z-stack imaging (Figure 2B). As can be seen in Figure 2B, cells were homogeneously distributed within the 160 µm depth of the scaffold investigated. The great cellular attachment in SA–GEL–GA has been attributed to gelatin, which naturally contains the Arg-Gly-Asp (RGD) ligands [18]. Cellular attachment to the RGD sequence has been shown to be mediated through interactions with integrins on cellular surfaces [19]. The presence of pores facilitates the transfer of nutrients as well as waste exudation through water as a medium and as such promotes cellular proliferation and tissue formation. 

**Figure 3:**
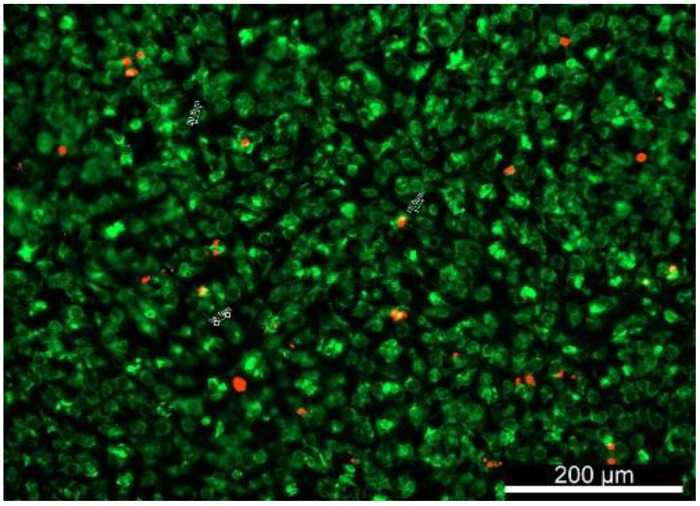
DiOC6(3)- and PI-stained bAChs within the SA–GEL–GA showing that the circular morphology was maintained by the cells at day 45, imaged one day after incubation, magnification 10×.

Chondrocytes in native AC span the different zones, and their numbers increase as a function of depth from 7000 to 24,000 cell/mm^3^ [20]. Chondrocytes have different shapes depending on which zone they exist in. They are flat in the superficial zone and round in the intermediate and deep zones, with sizes ranging from 10–15 µm [21]. The morphology of bAChs was qualitatively investigated via fluorescent imaging. Non-fixed scaffolds were imaged one hour after incubation with the dyes as described in Section 4.6, and the next day as shown in Figure 3. As can be seen in Figure 3, the cells maintained their circular morphology. Very few cells were stained red, additionally indicating that most cells were viable within the scaffold. Such morphology is typical of intermediate zone chondrocytes, which is to be expected, as the scaffold mimics the compressive modulus of the intermediate zone ranging from 50 kPa–250 kPa; the compressive modulus of the hydrogel was characterized in our previous work [15].

**Figure 4:**
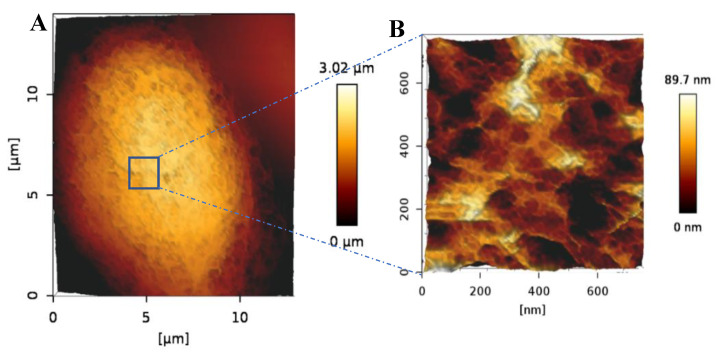
(**A**) AFM 3D height image of a bACh isolated from SA–GEL–GA taken under PBS showing the circular morphology of the cell. (**B**) 3D closeup scan area taken on top of the imaged cell showing the nanostructural details of the cell surface more closely.

To further explore the morphology of the bAChs grown in the SA–GEL–GA, AFM was used to image cells isolated from these gels at day 45. As can be seen in Figure 4, the cells maintained a circular morphology, which is an indicator of their native morphology. Fibrils can also be observed on the chondrocyte’s surface (Figure 4A). A smaller scan area on top of the chondrocyte reveals what appear to be pores in the cellular membrane (Figure 4B). Similar topographical AFM images of native human chondrocytes have been observed by Hsieh et al. [22].

**Figure 5:**
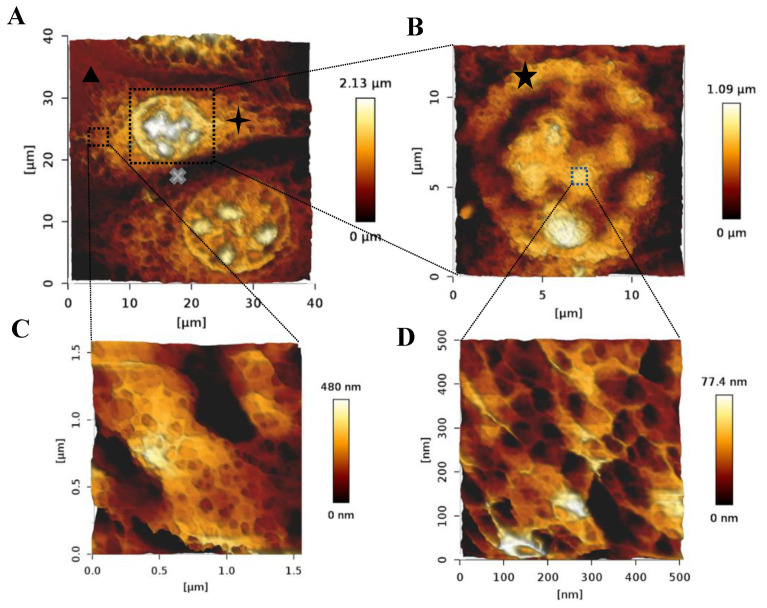
AFM 3D images taken in PBS of (**A**) a chondron isolated from SA–GEL–GA, (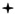) TM, (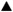) IM, and (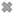) lacunae. (**B**) A chondrocyte showing the retained circular morphology and internal organelles, (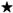) PCM. (**C**) A close-up on the nanostructure of the ECM surrounding chondrocytes within chondrons, and (**D**) the nanostructure of a region on top of the chondrocyte.

Chondrons are the basic metabolic units of AC tissues. They consist of chondrocytes and their surrounding PCM, IM, and TM [3]. While the exact function of such matrices is not very well understood, research has shown that they play a critical role in protecting chondrocytes from load-induced damage and regulating molecular transport between chondrocytes [4,23]. Chondrons have been shown to perform better in terms of matrix production in TE applications compared to single chondrocytes [24]. Intact chondrons were successfully isolated from SA–GEL–GA. Figure 5A shows an AFM image of a distinct chondron made of three chondrocytes and matrices surrounding them. It is worth noting that we have not found any AFM images of a whole chondron in the literature, and to the best of our knowledge this is a unique characterization. A close look at one of the chondrocytes embedded within a chondron is shown in Figure 5B. Figure 5C,D shows closeup images of the distinct structures observed with ECM surrounding cells within the chondron and on the surface of the cell, respectively. Figure 5D is very similar to the closeup image represented in Figure 4. Figure 5A,B also confirm the circular morphology expected for bAChs. As chondrocytes within chondrons are directly surrounded by PCM, TM, and IM, the matrices surrounding cells and observed in Figure 5 can be assumed to be the matrices mentioned above. The appearances of these matrices are similar to what Poole et al. observed using transmission electron microscopy (TEM) [25]. The size of the smaller cell in Figure 5A was 8.43 µm, with a PCM size of 2.22 µm, while the size of the bigger cell within the chondron (bottom) was 13.81 µm, with a PCM size of 2.01 µm. Similar dimensions for PCMs were reported by Chang et al. [26]. 

**Figure 6:**
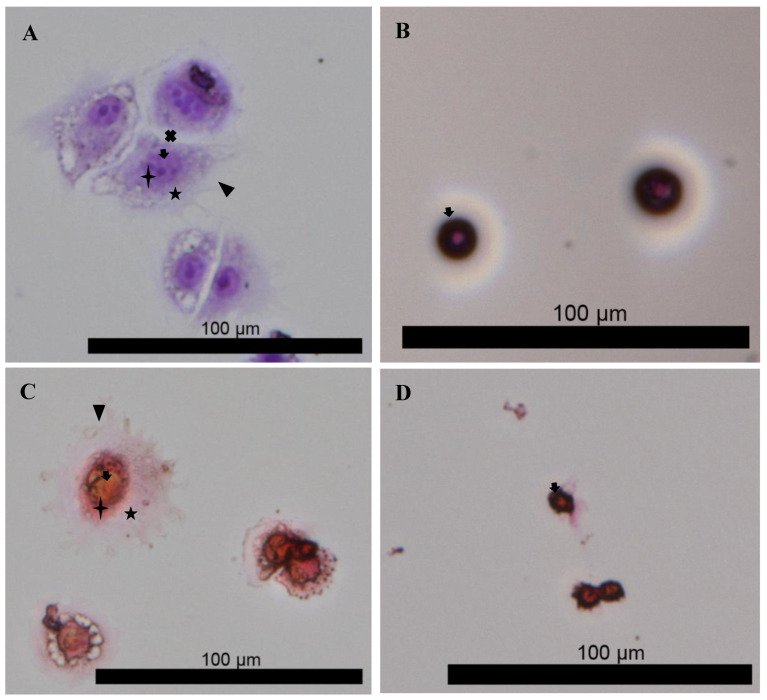
(**A**,**B**) Histological staining of GAG using DMMB for chondrons isolated from SA–GEL–GA showing the TM and IM, and (**B**) native chondrocytes at day 0 with smaller PCM and no TM or IM, respectively. (**C**,**D**) Histological staining of total collagen using Sirius red for chondrons isolated from SA–GEL–GA showing the PCM as the dark region around the cell and the TM and IM as the lighter regions further away, as well as that of a primary chondrocyte at day 0, respectively. 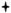 chondrocyte, 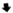 PCM, 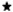 TM, 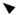 IM, and 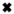 lacunae.

To further confirm this observation, the presence of a PCM, TM, and IM was investigated via histological staining (Figure 6A–D). Images shown in Figure 6A,C clearly show matrices around the isolated chondrons, as well as the lacunae, that is, the empty spaces between chondron constituents. Such matrices are absent from the images of enzyme digested native chondrocytes (Figure 6B,D). The histological images were analyzed by quantifying the total corrected intensity for both day 0 chondrocytes and day 45 chondrons. We found that chondrons produced 3.9-fold more glycosaminoglycans (GAGs) and 4.75-fold more collagen than day 0 chondrocytes (Figure 6A–D) (Supplementary Information S3). We observed that the PCM directly adjacent to chondrocytes within chondrons was enriched with more collagens (Figure 6C), while the TM and IM were more enriched with GAGs (Figure 6A). Such composition was expected, as the PCM directly surrounding chondrocyte cells is characterized by more collagen than that further away, while more GAG exist in the IM and TM [25,27]. Further analysis of the isolated PCM showed an enrichment with chondrogenic markers compared to day 0 chondrocytes. The transformation of chondrocytes into chondrons reduced the need to grow chondrocytes in aggregates or pellet cultures prior to their incorporation in a TE scaffold, as Li et al. observed less dedifferentiation when they used cellular aggregates compared to chondrocytes within hydrogels they developed [28].

### 2.3. Biochemical Analysis of DNA, GAG, and Collagen

**Figure 7:**
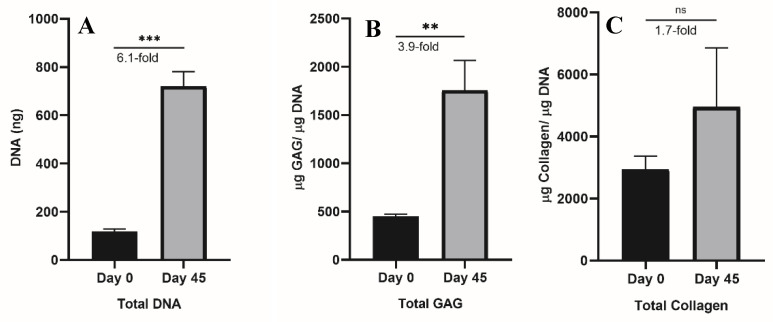
(**A**) DNA quantified using Quanti Fluor dsDNA for bAChs at day 0 and after 45 days of culture in SA–GEL–GA (n = 4, mean ± SEM, ** *p* < 0.01, *** *p* < 0.001). (**B**) Amount of GAGs quantified using DMMB assay normalized by DNA for chondrocytes at day 0 and day 45 cultured in SA–GEL–GA (n = 4, mean ± SEM, * *p* < 0.05). (**C**) Amount of collagen quantified using Sirius red assay normalized by DNA for chondrocytes at day 0 and day 45 cultured in SA–GEL–GA (n = 4, mean ± SEM, * *p* < 0.05, ns *p >* 0.05).

High cellular numbers are required for sufficient seeding in AC TE applications. These are usually acquired by the expansion of cells in a monolayer where a high number of cells can be achieved, albeit with the risk of a phenotype loss and dedifferentiation. An ideal alternative would be to induce the proliferation while maintaining a cartilage-specific phenotype [29] capable of producing key AC markers such as GAG and collagen. Chondrocytes grown in SA–GEL–GA exhibited high proliferation, as the amount of DNA was 6.1-fold higher than day 0 (Figure 7A). This confirms the results shown in the previous Section 3, as SA–GEL–GA supported chondrocytes’ attachment and migration. Chondrocytes grown in SA–GEL–GA produced 3.9-fold more GAG compared to day 0 (Figure 7B), and this is an indicator of a hyaline-like ECM deposition. Finally, no significant differences in total collagen production between chondrocytes grown in SA–GEL–GA and day 0 chondrocytes were found (Figure 7C). However, as mentioned in Section 2.2 (Figure S1A,B in Supplementary Information S3), when chondrons were compared to day 0 chondrocytes, the total collagen amount was 4.75-fold greater. This discrepancy was probably due to the high affinity of Sirius red dye to the gelatin present in the SA–GEL–GA, which in turn increased the blank reading. This could be one of the limitations of SA–GEL–GA gels. A more specific detection method could overcome this limitation, such as the use of collagen II antibodies for the detection of collagen II, for example. 

### 2.4. Immunofluorescence of Collagen I, Collagen II, Aggrecan, and CD44

**Figure 8:**
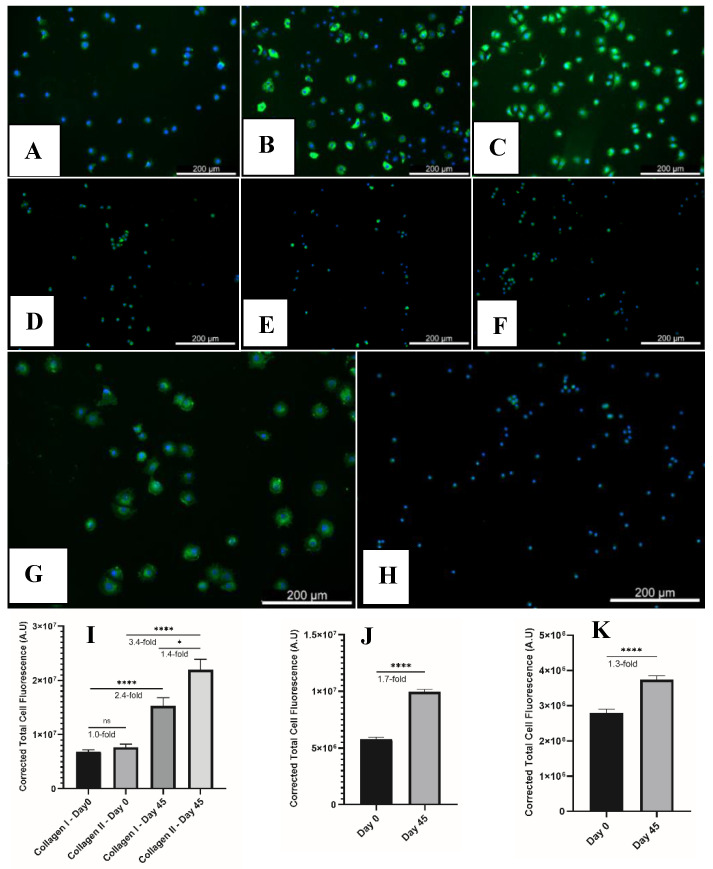
Immunofluorescence staining of collagen I, collagen II, and aggrecan for isolated chondrons (**A**–**C**, respectively) and day 0 primary chondrocytes (**D**–**F**, respectively). Immunofluorescence staining of CD44 for (**G**) isolated chondrons and (**H**) day 0 primary chondrocytes. Corrected total cell fluorescence of (**I**) collagen I vs. collagen II, (**J**) aggrecan, and (**K**) CD44. Images were all captured with 10× magnification.

In the chondron, the PCM is rich in different components such as collagen II, hyaluronan, and glycosaminoglycans, as well as collagen VI, which exists exclusively in the PCM [30]. To further characterize the chondrons isolated from SA–GEL–GA scaffolds, primary antibodies specific to collagen I, collagen II, aggrecan, and cluster of differentiation (CD44) were used to fluorescently detect the presence of their corresponding antigens (Figure 8A–H). Chondrons isolated from SA–GEL–GA produced more collagen II than collagen I, which indicates a retention of the chondrocyte phenotype (Figure 7A,B). The difference was quantified by calculating the corrected total cellular fluorescence (CTCF) for collagen I vs. collagen II. The value for collagen II was 1.44-fold higher than that for collagen I (Figure 8I). Primary chondrocytes at day 0 were stained for collagen I and collagen II as well and the fluorescence was quantified in the same way. The ratio of collagen II to collagen I was found to be 1.0. Chondrons isolated from SA–GEL–GA produced aggrecan, as shown in Figure 8C, that seemed to be localized on the cells as well as the TM and IM, which is similar to what we observed in DMMB histological staining (Figure 6A). When quantified, chondrons produced 1.7-fold more Aggrecan than day 0 native chondrocytes (Figure 8J). Finally, chondrocytes in the isolated chondrons expressed higher levels of CD44 than did native chondrocytes (1.3-fold) (Figure 8K). This was expected as CD44 is the cell receptor for hyaluronan in the hyaluronic acid–proteoglycan complex (HA–PG) [31]. The similarity in the chondrons’ immunofluorescence staining pattern between aggrecan (Figure 8C) and CD44 (Figure 8G) is explained by the fact that chondrocytes interact with aggrecan and hyaluronan through CD44 glycoprotein [32].

## 3. Conclusions

In this work, we have demonstrated the exceptional performance of SA–GEL–GA scaffolds for AC TE. SA–GEL–GA was not cytotoxic to bAChs and supported their proliferation while maintaining a circular morphology and producing hyaline-like ECM components such as GAGs and collagen. After 45 days of culture, bAChs grown in SA–GEL–GA created PCMs and ECMs and turned into chondrons that were enriched with collagen II, aggrecan, and CD44. The results presented in this work call for further explorations aimed at confirming that the SA–GEL–GA gel will produce similar exceptional results with human chondrocytes for potential use in MACT applications.

## 4. Materials and Methods

### 4.1. Materials

The following materials were purchased from Sigma-Aldrich (St Louis, MO, USA): (1-ethyl-3-(3-dimethylaminopropyl) carbodiimide hydrochloride) (EDC), 2-[4-(2-hydroxyethyl)piperazin-1-yl]ethane sulfonic acid (HEPES), alginic acid salt (SA), bovine serum albumin (BSA), calcium chloride (CaCl_2_), ciprofloxacin, dimethyl methylene blue (DMMB), dimethyl sulfoxide (DMSO), direct red 80, ethylenediaminetetraacetic acid (EDTA), gelatin 300 bloom from porcine skin (GEL), glacial acetic acid, glycine, guanidine hydrochloride, gum Arabic from acacia tree (GA), L-cystine HCL, N-hydroxy succinimide (NHS), normal goat serum, papain from papaya, paraformaldehyde 37%, picric acid saturated, propan-1-ol, sodium acetate, sodium hydroxide (NaOH), sodium chloride (NaCl), and Triton X-100. The following materials were purchased from Invitrogen (Carlsbad, CA, USA): aggrecan monoclonal antibody, Alexa Fluor 488 secondary antibody, calcein-AM, CD44 monoclonal antibody, collagen I monoclonal antibody, collagen II monoclonal antibody, Dulbecco’s minimum essential medium/Ham’s F-12 (DMEM/Ham’s F-12), phosphate-buffered saline (PBS), trypan blue, and TrypLE. Collagenase B was purchased from Roche (Basel, Switzerland). 3,3′-Dihexyloxacarbocyanine iodide (DiOC6(3)) was purchased from Enzo Lifesciences (Farmingdale, NY, USA). Propidium iodide (PI) was purchased from Alfa Aesar (Haverhill, MA). The Quanti Fluor dsDNA kit was purchased from Promega (Madison, WI, USA).

### 4.2. Scaffolds’ Preparation

SA–GEL–GA scaffolds were prepared as described before [15] with slight modifications. Briefly, known weights of SA, GEL, and GA pertaining to 5%, 10%, and 5% *w*/*v* respectively were mixed with warm deionized (DI) water maintained at 60 °C. The slurry was then mixed for five 12 s intervals using an Algimax II GX300 mixer (Holy Medical, People’s Republic of China). The slurry was loaded into a 60 mL syringe and extruded through a 25 G tapered plastic nozzle with cut end to ease extrusion. The gel was filled into 1 cm^3^ cubic molds and incubated in the fridge at 4–8 °C for 30 min. The scaffold cubes were then incubated in the fridge in 102 mM CaCl_2_ crosslinking solution in HEPES, pH 6.2 for one hour. The scaffolds were then washed with DI water and incubated for one hour in NHS-EDC solution made of 0.5 wt % EDC and 0.25 wt % NHS in the fridge. Finally, the scaffolds were washed and incubated in DI water at room temperature until use.

### 4.3. Primary Bovine Chondrocytes’ Isolation

Intact cow knees were obtained freshly postmortem from HEB store (San Antonio, TX, USA). Knees were washed with tap water to remove blood and hair, then with 70% ethanol prior to isolation in the biosafety cabinet. The joints were cut open using a #21 scalpel. The synovial fluid was then drained and cartilage pieces from the metacarpophalangeal joints were shaved using a #15 scalpel. Articular cartilage (AC) pieces were washed with PBS containing 1% ciprofloxacin three times. AC pieces were digested overnight in a 100 mg/mL collagenase B digestion medium made of DMEM/Ham’s F-12, 3% FBS, and 2% ciprofloxacin. The digested tissues were then filtered using a 40 µm Steriflip filter (Millipore Sigma, St Louis, MO, USA), and bovine articular chondrocytes (bAChs) were collected and washed three times with expansion medium. Cells were then counted using trypan blue exclusion with a Countess II (ThermoFisher Scientific, Waltham, MA). bAChs were then cryopreserved in 5% DMSO/95% FBS solution and kept at −84 °C until use.

### 4.4. Cell Culture

Prior to cellular seeding, scaffolds were incubated in 70% ethanol in a biosafety cabinet at room temperature for one hour to sterilize them. Scaffolds were then washed with PBS three times and then incubated in a humidified incubator at 37 °C and 5% CO_2_ in an expansion medium made of DMEM/Ham’s F-12 supplemented with 10% FBS and 1% Ciprofloxacin until seeding. Media were removed and scaffolds were seeded with bAChs at a density of 100,000 cells/ cm^3^. Cultures were maintained for 45 days with media change every other day.

### 4.5. Cytotoxicity Evaluation

The cellular viability of bAChs grown in SA–GEL–GA was determined using a PrestoBlue viability assay according to the manufacturer’s protocol. Briefly, a specific volume equal to 10% of the cell culture medium was added to the scaffold cultures and cells grown on tissue culture plastic (TCP) and incubated in a humidified incubator at 37 °C and 5% CO_2_ for 1 h, the reduction of PrestoBlue by the cells was then measured using a Cytation 5 multiplate reader (BioTek Instruments Inc., Winooski, VT, USA). Fluorescence was determined at excitation/emission = 535/615 nm.

To qualitatively visualize the viable cells inside SA–GEL–GA, the scaffold cultures were incubated in a solution made of 2 µM calcein AM and 5 µM propidium iodide for 1 h at 37 °C and 5% CO_2_. Live cells stain green and can be visualized with a green fluorescence protein (GFP) filter cube, excitation/emission = 469/525 nm. In comparison, dead cells stain red and can be visualized with a Texas red filter cube, excitation/emission = 586/647 nm). The live/dead fluorescent images were visualized using a Cytation 5 multiplate reader.

### 4.6. Cellular Morphology and Distribution

To observe the cellular morphology of chondrocytes grown in SA–GEL–GA, the cells’ membranes were stained using DiOC6(3) lipophilic fluorescent dye that stains green and which can be visualized with a GFP filter cube, excitation/emission = 469/525 nm. 5 µM of propidium iodide was used as a nuclear counter stain that stains red and can be visualized with a Texas red filter cube, excitation/emission = 586/647 nm. Two dimensional (2D) images were acquired using a Cytation 5 multiplate reader, and three dimensional (3D) rendered images were acquired using a Leica DM800i Confocal Microscope (Leica Microsystems, Bannockburn, IL, USA).

### 4.7. Biochemical Analyses of DNA, GAG, and Collagen

In preparation for analysis, SA–GEL–GA-seeded scaffolds, day 0 chondrocytes, as well as empty SA–GEL–GA scaffolds as a blank were digested overnight at 60 °C in papain digestion buffer (Supplementary Information S1.1). DNA analysis was done using a Quanti Fluor dsDNA kit according to manufacturer’s protocol. Briefly, a 20 µL sample was added to 200 µL of Quanti Fluor dsDNA dye prepared in 1× TE buffer. The fluorescence was detected using a Cytation 5 multiplate reader against a blank of TE buffer for day 0 chondrocytes at an excitation/emission = 504/531 nm. Fluorescence was quantified using a standard curve prepared with λ-DNA.

The amount of glycosaminoglycans (GAGs) produced by the cultured bAChs was determined using DMMB assay as previously described by Barbosa et al. [33]. Briefly, the papain-digested cell-laden hydrogel was centrifuged at 14,000 rpm for 10 min, then 50 µL of the supernatant was added to 1 mL DMMB dye solution in an Eppendorf tube (Supplementary Information S1.2). The mixture was shaken for 30 min at room temperature, then centrifuged at 12,000 rpm for 10 min. The supernatant was discarded, and the GAG-dye complex pellet was released with 1 mL of DMMB decomplexation solution (Supplementary Information S1.3). The absorbance was measured at 656 nm using a Cytation 5 multiplate reader and concentrations were evaluated using a standard curve of chondroitin sulfate (Biocolor Ltd., Carrickfergus, U.K.). Empty scaffolds were used as a blank.

To determine the total collagen produced by bAChs in SA–GEL–GA, Sirius red assay was used as described previously by Walsh et al. [34]. Briefly, 50 µL of papain-digested supernatant was added to 1 mL of Sirius red dye solution and shaken for 30 min at room temperature (Supplementary Information S1.4). The mixture was then centrifuged at 12,000 rpm for 10 min. The supernatant was then discarded, and the pellet was washed with 750 µL of cold acid salt wash (Supplementary Information S1.5) and centrifuged at 10,000 rpm for 5 min. The collagen-dye complex pellet was then released using 1 mL Sirius red dye release solution (Supplementary Information S1.6). Absorbance was then measured at 550 nm using a Cytation 5 multiplate reader. Concentration was evaluated using a standard curve of rat tail collagen I (Biocolor Ltd., UK).

### 4.8. Histological Staining

To visualize total collagen and total GAGs produced by chondrons isolated from SA–GEL–GA and compare them to day 0 bAChs, chondrons were isolated from the scaffolds by incubating the scaffolds in TrypLE for 30 min at 37 °C. The cell-laden scaffold was then vortexed to help it disintegrate. Chondrons were collected by filtering through a 40 µm Steriflip filter. Isolated chondrons and day 0 chondrocytes were seeded on 35 mm petri dishes at a seeding density of 300,000 cells/dish. Both were allowed to adhere for 1 h at 37 °C prior to fixing with a 4% paraformaldehyde solution. To histologically stain for total collagen, chondrons and day 0 chondrocytes were incubated in Sirius red staining solution for 30 min, then washed with 1% acetic acid and imaged using a Cytation 5 multiplate reader. To stain for GAGs, chondrons and day 0 chondrocytes were incubated in DMMB staining solution for 30 min then washed with DI water and imaged using a Cytation 5 multiplate reader. Corrected total cellular intensity was evaluated as described in Supplementary Information S2.

### 4.9. Immunofluorescence Staining

To qualitatively visualize the proteins produced by chondrons isolated from SA–GEL–GA compared to day 0 chondrocytes, isolated chondrons and day 0 chondrocytes were seeded on a glass-bottomed 96-well plate at a density of 20,000 cells/well. They were allowed to adhere for 1 h at 37 °C prior to fixing with a 4% paraformaldehyde solution. They were then blocked with a blocking buffer (Supplementary Information S1.7) for 1 h. After blocking, cells and chondrons were incubated in primary antibody solution made with antibody dilution buffer (Supplementary Information S1.8) for 24 h in the fridge. The primary antibody dilutions were as follows: collagen II 1:100, collagen I 1:100, aggrecan 1:50, and CD44 1:100. Cells and chondrons were washed with PBS three times then incubated with Alexa Fluor 448 secondary antibody at a dilution of 1:500 for 1 h. The nuclei were then counterstained with DAPI for five minutes. Immunofluorescence intensity was imaged using a Cytation 5 multiplate reader. To compare collagen II to collagen I fluorescence, aggrecan, and CD44, corrected total cellular fluorescence [35] was evaluated as described in Supplementary Information S2.

### 4.10. Atomic Force Microscopy (AFM) Imaging

Chondrons were imaged after fixation for cellular phenotype visualization, and after histological staining for chondrons’ structures visualization using AFM. AFM imaging was carried out using a NanoWizard IV AFM (JPK Instruments, Bruker, Billerica, MA, USA) with the CellHesion ™ module, which allows for a large z-range (100 µm) scanning. The AFM head was mounted on a Zeiss Axio Observer 3 Inverted Fluorescence Microscope (Carl Zeiss, Göttingen, Germany). All images were acquired in PBS. Images were acquired using a silicon nitride cantilever with a resonance frequency of 23kHZ and nominal spring constant of 0.12 N/m (SNL-10 probe, Bruker, Billerica, MA, USA). QI™ imaging mode was used to acquire AFM height images of 128 × 128 pixels with a z-length of 4.4 µm and a trigger set point of 3 nN. 3D topographical images were produced using NanoWizard data analysis software (JPK Instruments, Bruker, Billerica, MA, USA).

### 4.11. Statistical Analysis

Statistical significance was determined using an independent Student’s *t*-test using GraphPad Prism (GraphPad Software, San Diego, CA, USA). Data are represented as mean ± standard error of the mean (SEM), and a *p* value of < 0.05 was considered statistically significant.

## Data Availability

The data presented in this study are available on request from the corresponding author. The data are not publicly available because they present only a subset of data generated and we retain the right to data until all of it has been published.

## Supplementary Materials

The following supporting information can be downloaded at: https://www.mdpi.com/article/10.3390/gels8020090/s1, Section S1: Materials Recipes, Section S2: Corrected Total Cellular Reflectance/Fluorescence Quantification, Figure S1: Corrected Total Reflectance Comparison of Histological Staining.
