# Supplementary material for: From Chondrocytes to Chondrons, Maintenance of Phenotype and Matrix Production in a Composite 3D Hydrogel Scaffold
Source: Gels. 2022 Feb 2;8(2):90. doi: 10.3390/gels8020090 (PMC8871571; doi:10.3390/gels8020090)
Supplement: Supplementary file 1 [file gels-08-00090-s001.zip › gels-1538061-supplementary.pdf]

Supplementary Information for

From Chondrocytes to Chondrons, Maintenance of Phenotype and Matrices Production in a Composite 3D Hydrogel Scaffold

Mahmoud Amr [1], Alia Mallah [1], Samina Yasmeen [2], Bernard Van Wie [3], Arda Gozen [4], Juana Mendenhall [2], and Nehal I. Abu-Lail [1,*].

1 Department of Biomedical Engineering and Chemical Engineering, The University of Texas at San Antonio, San Antonio, Texas, 78249; mahmoud.amr@utsa.edu, alia.mallah@utsa.edu, Nehal.abu-lail@utsa.edu

2 Department of Chemistry, Morehouse College, Atlanta, Georgia, 30314; samina.yasmeen@morehouse.edu, juana.mendenhall@morehouse.edu

3 Gene and Linda Voiland School of Chemical Engineering and Bioengineering, Washington State University, Pullman, Washington, 99164-6515; bvanwie@wsu.edu

4 School of Mechanical and Materials Engineering, Washington State University, Pullman, Washington, 99164-2920; arda.gozen@wsu.edu

* Correspondence: Nehal.abu-lail@utsa.edu; One UTSA Circle, Department of Biomedical Engineering and Chemical Engineering, The University of Texas at San Antonio, San Antonio, Texas 78249

S1. Materials Recipes

S1.1. Papain digestion buffer

The digestion buffer was made of 0.2 M Sodium Phosphate Buffer (Na_2HPO_4 – NaH_2PO_4), at pH of 6.4 and was composed of:

- 8 mg/mL Sodium Acetate
- 4 mg/mL EDTA
- 0.8 mg/mL L-Cystine HCL

After dissolving all the solid powder in the buffer, 50 µL/mL of Papain from papaya containing (0.1 mg enzyme) was added to make up the final volume. Papain digestion buffer is good for 7 days at 4 °C.

S1.2. DMMB dye solution

Dissolve 0.016 mg/mL DMMB, 3.04 mg/mL glycine, 1.6 mg/mL, and 0.095 mL/mL 0.1 M acetic acid. Make up the volume to the volume needed with DI water. Filter the solution using 0.22 µm, and protect from light, store for 1 month at room temperature.

S1.3. DMMB decomplexation solution

The decomplexation buffer was made of 50 mM sodium acetate solution (pH 6.8) containing 10% propan-1-ol and was used to prepare 4 M guanidine hydrochloride. The solution is stable for 4 months at room temperature.

S1.4. Sirius red dye solution

Dissolve 0.1 g Direct Red 80 in 100 mL saturated picric acid, mix gently till it’s all dissolved. Cover from light and store at room temperature up to 1 month.

S1.5. Acid salt wash

The acid salt wash was made of 1 mL glacial acetic acid in 200 mL DI water.

S1.6. Sirius Red dye release solution

The dye release solution was made of 0.1 M NaOH. It can be stored up to a month at room temperature.

S1.7. Blocking buffer solution

The antibody blocking buffer was made of 5% normal goat serum in 0.3% Triton X-100 diluted in PBS. It can be stored up to 1 month at 4 °C.

S1.8. Antibody dilution buffer

The antibody dilution buffer was made of 1% BSA in 0.3% Triton X-100 diluted in PBS. It can be stored up to 1 month at 4 °C.

S2. Corrected Total Cellular Reflectance/Fluorescence Quantification

Following the protocol published by Luke Hammond [34], to quantify the corrected total cellular fluorescence (CTCF) using ImageJ software (http://rsbweb.nih.gov/ij/download.html):

- Open ImageJ and choose import the image you want to analyze (the channel for the protein of interest)
- Select: “Analyze>Set Measurements”
- Check the selection of the following: Area Integrated Intensity and Mean Grey Value

- In the image select an empty area on the background around the cell of interest using the selection tool
- Then click “Analyze>Measure”
- A measurement table will appear
- Repeat the process for all the cells you want to quantify CTCF for, in the end you will have a list of background integrated intensity values that you will average and use for the calculations.
- Now using the freehand selection tool select the area of each cell of interest and hit “Analyze>Measure” after each cell selection.
- In the end you will have a table of values for all the cells quantified
- The CTCF formula is : $CTCF = Integrated\ Density - (Area\ of\ selected\ cell \times average\ of\ integrated\ density\ for\ background\ readings)$
- Report the values as Corrected Total Cellular Fluorescence (Arbitrary Unit or A.U.)
- The same can be done for histological images for RGB images, you can analyze and compare one of the 3 channels (in our case we analyzed the RED).
- Quantification can also be automated using BioTek’s Gen 5.0 software (BioTek Instruments Inc, Winooski, VT) if you have the software license.

S3. Corrected Total Reflectance Comparison of Histological Staining

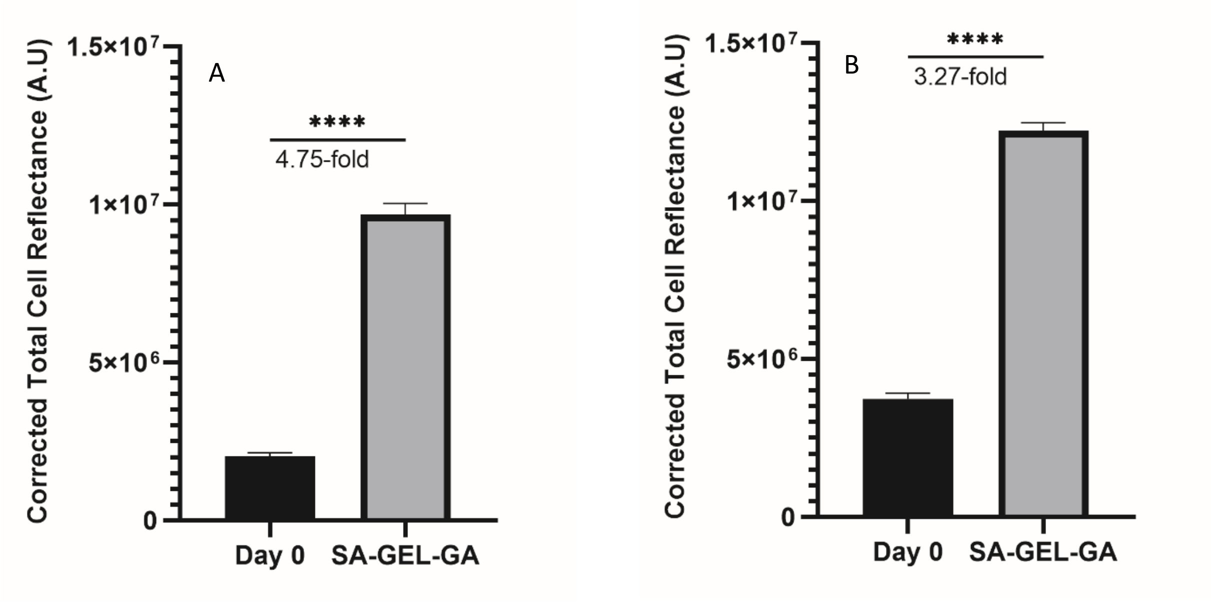

Figure S1. Corrected Total Cell Reflectance for A) Sirius Red histological staining of chondrons isolated from SA-GEL-GA vs day 0 chondrocytes, B) DMMB histological staining of chondrons isolated from SA-GEL-GA vs day 0 chondrocytes, data is presented as Mean±SEM, **** $P < 0.0001$, $n = 3$.
